# Multiple Imputation of Missing Composite Outcomes in Longitudinal Data

**DOI:** 10.1007/s12561-016-9146-z

**Published:** 2016-04-05

**Authors:** Aidan G. O’Keeffe, Daniel M. Farewell, Brian D. M. Tom, Vernon T. Farewell

**Affiliations:** 1 Department of Statistical Science, University College London, Gower St., London, WC1E 6BT UK; 2 Institute of Primary Care and Public Health, Cardiff University School of Medicine, Neuadd Meirionnydd, Heath Park, Cardiff, CF14 4YS UK; 3 MRC Biostatistics Unit, Cambridge Institute of Public Health, Forvie Site, Robinson Way, Cambridge Biomedical Campus, Cambridge, CB2 0SR UK

**Keywords:** Composite outcome, Linear increments, Longitudinal data, Missing data, Multiple imputation

## Abstract

In longitudinal randomised trials and observational studies within a medical context, a composite outcome—which is a function of several individual patient-specific outcomes—may be felt to best represent the outcome of interest. As in other contexts, missing data on patient outcome, due to patient drop-out or for other reasons, may pose a problem. Multiple imputation is a widely used method for handling missing data, but its use for composite outcomes has been seldom discussed. Whilst standard multiple imputation methodology can be used directly for the composite outcome, the distribution of a composite outcome may be of a complicated form and perhaps not amenable to statistical modelling. We compare direct multiple imputation of a composite outcome with separate imputation of the components of a composite outcome. We consider two imputation approaches. One approach involves modelling each component of a composite outcome using standard likelihood-based models. The other approach is to use linear increments methods. A linear increments approach can provide an appealing alternative as assumptions concerning both the missingness structure within the data and the imputation models are different from the standard likelihood-based approach. We compare both approaches using simulation studies and data from a randomised trial on early rheumatoid arthritis patients. Results suggest that both approaches are comparable and that for each, separate imputation offers some improvement on the direct imputation of a composite outcome.

## Introduction

When study patients are followed longitudinally, many patient-specific outcomes may be collected over time. A composite measure that combines these outcomes is often used to provide an overall assessment of a patient’s condition. For example, for clinical trials in rheumatoid arthritis, the American College of Rheumatology 20 % composite outcome, denoted ACR20, combines information on several variables concerning disease severity into a binary indicator based on which and how many of these variables have demonstrated 20 % reductions over time. Whilst it is recommended that all the separate components of the ACR20 be reported in trial results, a focus on ACR20 is common.

Some or all of the outcome variables that contribute to a composite outcome may be missing at certain time points. Whilst it is simple to focus solely on a ‘complete case’ analysis, based only on data for patients who have completely observed data at one or more time points, multiple imputation is widely recognised as useful to guard against biased inferences, particularly those owing to unrepresentative complete case data [10, 14, 17, 18, 20]. Multiple imputation, first introduced by Rubin [15] and described extensively in [12], generally involves the assumption of a structure for the relationship between the observed and the missing data, the fitting of this model to the ‘complete case’ responses and the use of the fitted model to predict outcomes where missing values exist. The model from which imputations are drawn is usually fully parametric and can be fitted using maximum likelihood (ML) methods.

In this work, we examine models for the multiple imputation of missing composite outcomes in longitudinal studies, where the time points at which observations are made are fixed by design. Standard multiple imputation procedures are investigated for directly imputing the composite outcome and for indirect imputation of the composite outcome through imputation of its component measures. In addition, we introduce methods to base multiple imputation on linear increments estimation [6]. Linear increments (LI) methods for imputation are compared with more standard multiple imputation procedures. To our knowledge, no work has explored multiple imputation in longitudinal data using an LI modelling approach.

For illustration, we focus on imputation of the ACR20 based on data from the CARDERA longitudinal randomised trial. The trial was designed to provide 24 months of follow-up and therefore we take the parameter of inferential interest to be the probability of the event {ACR20 at 24 months = 1}. This paper is organised as follows: in Sect. 2 we outline the CARDERA trial and provide a definition of ACR20. Section 3 provides a description of the two types of multiple imputation that we consider: an ML-based method and an LI method. In Sect. 4, we outline the multiple imputation processes for the CARDERA trial data. In Sect. 5, we perform comparisons of the imputation approaches based on various simulated missingness scenarios within the CARDERA trial dataset. A short example applying the imputation methods to the actual missing data in the CARDERA trial is presented in Sect. 6. A discussion is provided in Sect. 7.

## The CARDERA Trial

The Combination Anti-Rheumatic Drugs in Early Rheumatoid Arthritis (CARDERA) trial recruited patients with early rheumatoid arthritis, seen at routine rheumatology outpatient clinics across England and Wales. The trial is described extensively in Choy et al. [2] and was devised as a two-year double-blind randomised controlled trial with the aim of determining the relative benefits of various combinations of disease-modifying anti-rheumatic drugs (DMARDs) and glucocorticoids. Patients were randomised to one of four treatment groups in a $2 \times 2$ factorial design and were followed up every 6 months for two years. Baseline information was collected, and outcomes were measured at baseline and at each follow-up visit. The trial recruited 467 patients (142 males and 325 females). The aim of the trial was to examine whether or not combining methotrexate with glucocorticoids and/or ciclosporin in early rheumatoid arthritis reduced the proportion of patients who developed new radiological joint damage within two years. Here, for illustration, we focus on a secondary composite outcome, ACR20, which the trial reported as not differing significantly amongst the treatment groups after 24 months.

The ACR20 takes a value of 0 or 1 at any time point, depending on the changes in the values of various patient-specific measurements from baseline. Two measurements of primary importance are the tender joint count (TJC) and the swollen joint count (SJC), each being a count of joints across 28 joint locations. The ACR20 takes the value 1, representing patient improvement, if at 20 % reductions in both TJC and SJC are observed, together with at least a 20 % reduction in at least three of the following five variables:

- Erythrocyte sedimentation rate (ESR);
- Physician global assessment of disease activity (AGA)—a visual analogue scale (VAS) ranging from 0 to 100 where 0 and 100 represent the least and greatest activities, respectively;
- Patient global assessment (PGA) of disease activity—a VAS from 0 to 100 where 0 and 100 represent the least and greatest activities, respectively;
- Patient assessment of pain (Painscore)—a VAS from 0 to 100 where 0 and 100 represent the least and greatest pains, respectively.
- Health Assessment Questionnaire (HAQ)—a measure of functional disability providing a score from 0 to 3, where 3 represents the most severe disability.

Having described the CARDERA trial and our composite outcomes of interest, we turn our attention to statistical models that might be used for the ACR20 outcome at 24 months.

## Statistical Models for Patient Outcomes

We consider suitable statistical models that may be used for the multiple imputation of ACR20. Specifically, we focus on the modelling of each component used in the calculation of ACR20, using two methods: maximum likelihood estimation (MLE) and LI modelling.

ACR20 is a composite outcome and, as such, it may perhaps be more appealing to investigate its individual components and therefore specify the distributions of its component outcomes rather than rely on a single binary model. However, we recognise that researchers will usually be interested in making inferences for the composite outcome ACR20 as well as for the component outcomes. The separate imputation of both missing ACR20 values and missing component outcome values, which, in combination, specify another imputed ACR20 value, may lead to conflicting imputed composite outcomes. In addition, specification of correct functional forms or models for component outcomes might sometimes be easier, conceptually, than assuming a particular functional form or model for a composite outcome. We outline models that may be used for the patient-level prediction of components at 24 months. The predicted values of components may then be combined to produce an overall estimate of ACR20 at 24 months.

We define $Y_{ij}$ to be a directly recorded (non-composite) single outcome of interest in the CARDERA trial for the *i*th patient at time points $t_{j} \in \{t_{1}, t_{2}, t_{3}, t_{4}, t_{5}\}$, representing baseline and four 6 monthly follow-up times, such that $\mathbf {Y}_{i}$ is a vector of outcomes across all time points for the *i*th patient and $\mathbf {Y}$ denotes the complete set of outcomes across all patients with deaths ignored for notational convenience. We aim to model $\mathbf {Y}$ based on complete case data from each time point (i.e. those patients for whom $Y_{ij}$ is known at time $t_{j}$) and impute data as appropriate. As we shall discuss, for convenience, we assume initially that the individual outcomes at the same time point are independent. Furthermore, we assume that the distribution of $Y_{ij}$ is a member of the exponential family of distributions, the probability density/mass function of which is given by

1 $$\begin{aligned} f_{Y_{ij}}(y_{ij}|\nu _{ij},\phi ) = \exp \left\{ \frac{(y_{ij}\nu _{ij}-b(\nu _{ij}))}{\phi }-c(y_{ij},\phi )\right\} , \end{aligned}$$

with dispersion parameter $\phi \in \Phi \subseteq (0,\infty )$, canonical parameter $\nu _{ij}$ and known functions *b*(.) and *c*(., .). Usually, we assume that $\mathbb {E}(Y_{ij}) = \mu _{ij}$ and that $\mu _{ij}$ is a function of $\nu _{ij}$ only. To model a dependence of $\mu _{ij}$ on $\mathbf {x}_{ij}$, a vector of known explanatory variables for the *i*th patient available at the time point $t_{j}$, we assume that there exists a link function *h*(.) such that

$$\begin{aligned} h(\mu _{ij}) = \varvec{\beta }_{j}^{T}\mathbf {x}_{ij}. \end{aligned}$$

To estimate the parameter $\varvec{\beta }_{j}$, we consider MLE and LI methods.

### Maximum Likelihood Estimation

Based on the probability density/mass function of $Y_{ij}$ (Eq. 1), a likelihood function can be defined and maximised using iterative optimisation methods to provide parameter estimates $\hat{\varvec{\beta }}_{j}$ together with their associated standard errors. A ML model, fitted on complete cases, may be used as a model from which imputed values may be drawn at random, transformed using the inverse function $h^{-1}(.)$ and imputed in place of missing observations. Such models are fully parametric.

### Linear Increments Estimation

LI methodology was introduced by Farewell [6, 7]. An LI approach makes different assumptions about the dropout process than those in traditional missing at random-based analyses [13]. In particular, dropout can depend on an *unobserved* random walk random effect (a martingale), but not on future events (dropout is a predictable process). Another important difference is that LI only specifies a model for the mean of the outcomes, and no further assumptions at all are needed regarding the dropout process.

Suppose we define

$$\begin{aligned} \Delta Y_{ij} = Y_{ij} - Y_{ij-1} \end{aligned}$$

to be the increment for the outcome $Y_{i}$ (for the *i*th patient) between the time points $t_{j-1}$ and $t_{j}$. We think of the successive observations $Y_{i1}, Y_{i2}, \ldots , Y_{i5}$ as realised values of a continuous time stochastic process $\{Y_{it},t\in \mathcal {T}\}$ for an ordered time set $\mathcal {T}$. We define, for each patient, two further stochastic processes over $\mathcal {T}$:

- A multivariate explanatory variable process: $\{X_{it}, t\in \mathcal {T}\}$
- A mean-zero martingale error process: $\{\epsilon _{it}, t\in \mathcal {T}\}$

and denote $\mathcal {F}_{t-}$ to be the history of of the outcome, explanatory variable and error processes up to time *t*.

The expected value of the incremental changes in $\mathbf {Y}_{i}$ from $t_{j-1}$ to $t_{j}$, conditional on the history $\mathcal {F}_{t_{j}-}$, that includes previous responses and covariate history, may be written

$$\begin{aligned} \mathbb {E}(\Delta Y_{ij}|\mathcal {F}_{t_{j-}}) = X^{T}_{i,j-1}\varvec{\beta }_{j}. \end{aligned}$$

with $\varvec{\beta }_{j}$ denoting a suitable vector of explanatory variable effects and intercept term.

Despite the specification of a form for the expected value of the outcomes $\mathbf {Y}$, an MLE-based method is not used to fit LI models. Instead, a non-parametric method is used. Although not necessary for LI methods generally, we make an assumption of monotonic missingness. In other words, once an outcome is missing at one time point, it is also missing at all subsequent time points. Thus, we assume missingness arises through patient drop-out/withdrawal.

We define $\bar{Y}_{j}^{(l)}$ to be the sample mean of the outcome *Y* at time $t_{j}$ calculated using observed values of the outcome from those patients who have at least *l* non-missing observations ($l \ge j$). Using this notation, the LI estimate of the population mean outcome, $\mu _{j}$, at time $t_{j}$ is:

2 $$\begin{aligned} \tilde{\mu }_{j} = \sum _{l=2}^{j}\left( \bar{Y}^{(l)}_{l}-\bar{Y}^{(l)}_{l-1}\right) +\bar{Y}_{1}^{(1)} \end{aligned}$$

where these estimates may be allowed to depend on patient-level explanatory variables through a regression formulation. Here, $\tilde{\mu }_{j}$ is constructed as the average outcome value at the first time point plus the sum of the average incremental outcome changes up to $t_{j}$. Thus, the main requirement for the LI method is that increments must be representative of the general population which is a weaker assumption that that for a complete case analysis which requires that observed outcomes must be representative.

Generalised estimating equation (GEE) [21] software can be used to fit the models in cases where the number of time points at which measurements are made is relatively small [8]. Essentially, the LI method is implemented by adopting a fixed working correlation structure such that $\text {Corr}(Y_{ih}, Y_{ij}) = \text {min}(h, j)$. This covariance structure corresponds to setting the working correlation matrix to be a generalised inverse of the singular matrix of ones (i.e. the $n \times n$ singular matrix such that all entries of the matrix are equal to one). At first sight it may appear that an LI analysis is analogous to the analysis of change scores, commonly used in randomised trials [19]. With both methods, a difference in expected outcomes is modelled, although the linear increments approach involves the modelling of successive changes between subsequent time points whereas a change score analysis typically involves modelling a change since baseline.

Values may be drawn at random from models fitted by LI, transformed back to the same scale as the outcome and then imputed in place of missing observations, given a set of explanatory variables *X*. LI methods have been used to account for multivariate missing outcomes in longitudinal data [1, 9] although, to our knowledge, no work has used LI methods for multiple imputation, generally or specifically for missing composite outcomes.

## Multiple Imputation

We fitted models using both MLE and LI based on the complete observations at each time point for ACR20 and for each of the outcomes involved in its definition. The outcomes used to calculate ACR20 would be expected to be correlated within the same individual. Whilst a multivariate distribution for these seven outcomes could be considered, given the differing nature of these outcomes, the specification of such a multivariate distribution would be difficult. Alternatively, conditional distributions for the individual outcomes over time (for example, a conditional distribution for PGA at one time point given the change in TJC and SJC since the previous time point) could be considered. More directly, multiple imputation with chained equations can be used to reflect dependencies and this will be used in Sect. 6 for comparison purposes. However, here we want to focus primarily on the comparison of ML- and LI-based multiple imputation methods. Therefore, for simplicity, we make the assumption that the outcomes at the same time point, conditional on their respective histories, are independent within an individual and use fitted models for the marginal distribution of each outcome from which to draw imputations. Clearly, this assumption of independence may not always be correct and not accounting for correlation amongst outcomes may reduce the power of the multiple imputation approach. However, we aim for the imputation approach to be easy to implement and understand, which may not be the case if the modelling of putative correlation structures is introduced. All imputation models account for the past history of the outcome.

Consistent with the majority of the CARDERA data, we assume that when one outcome is missing at a particular time point, then all outcomes are missing. Thus, it would not be the case that a known outcome value at a time point could provide information on the likely value of a missing outcome at the same point for the same individual. This assumption is consistent with the notion that missingness arises due to patient drop-out or study withdrawal. We recognise that this assumption would not always be appropriate but we adopt it for computational simplicity in our simulations.

We assumed that TJC and SJC, joint counts from 0 to 28, are binomially distributed, which we model in terms of the empirical logit [5] with a normal approximation. The other outcomes involved in the calculation of ACR20 (PGA, AGA, Painscore, ESR and HAQ) are assumed, for simplicity, to be normal random variables, upon suitable transformations. The distributions of AGA, PGA and Painscore were truncated to lie in the interval [0, 100], HAQ to lie in the interval [0, 3] and ESR to lie in the interval [1, 200]. For each outcome (other than TJC and SJC), a square root transformation was used. This is because some of the variables contained a large number of values close to the lower limit of zero and hence displayed positive skewness in their distribution. A square root transformation helped to make an assumption of normality more appropriate for these variables.

For the multiple imputation process, models estimated by both MLE and LI are used to predict the outcomes of interest. Where an outcome value is missing, a new value is drawn from the appropriate model (conditioning on the explanatory variables from the patient) $M \in \mathbb {N}$ times. In doing so, *M* ‘imputed’ datasets are created. The quantities of interest (e.g. parameter estimates and associated standard errors) can then be computed by combining analyses from each of the imputed datasets following Rubin’s rules [16].

In the original CARDERA trial, the 467 patients were randomised to one of four treatment groups: methotrexate (MTX) only (117 patients), ciclosporin (CSP) and MTX (119 patients), prednisolone (PDN) and MTX (115 patients) and MTX, CSP and PDN (116 patients). Henceforth, these treatment groups are known as ‘None’, ‘CSP’, ‘PDN’ and ‘Both’ with these names describing the combination of CSP and PDN that each group was prescribed in addition to MTX.

We use treatment group as an explanatory variable along with the transformed outcome at the previous time point. The ML and LI imputation models at time $t_{j}$ are summarised as,

3 $$\begin{aligned} {\mathbb {E}}(g(Y_{ij})) = \alpha _{j}+\beta _{j}g(y_{ij-1})+{\varvec{\gamma }}_{j}^{T}{\mathbf {x}}_{i} \end{aligned}$$

and

$$\begin{aligned} \mathbb {E}(g(Y_{ij})-g(Y_{ij-1})) = \delta _{j}+\eta _{j}g(y_{ij-1})+\varvec{\omega }_{j}^{T}\mathbf {x}_{i} \end{aligned}$$

respectively where $\mathbf {x}_{i}$ denotes the set of explanatory variables relating to treatment group. The vectors $(\alpha _{j},\beta _{j},\varvec{\gamma }_{j}^{T})^{T}$ and $(\delta _{j},\eta _{j},\varvec{\omega }_{j}^{T})^{T}$ are parameter vectors to be estimated. The use of of transformed outcome data on the right hand side of these regression equations, to incorporate past history, is not required and the use of untransformed data in the linear predictor is also commonly used for this purpose. Other choices for extending the increments model to discrete outcomes are possible but we restrict ourselves to a linearised outcome in this paper. Algorithms for the multiple imputation methods and associated modelling assumptions are described in Appendix.

## Comparisons Based on Simulated Missingness

### Missingness in the CARDERA Trial

In this section, we compare the imputation approaches described in Sects. 3 and 4 using data from the CARDERA trial. We use $\text {ACR}^{(24)}20$ to denote the ACR20 value at 24 months, with $\mathbb {P}(\text {ACR}^{(24)}20 = 1)$ being the parameter of interest. Of the 467 randomised patients in the CARDERA trial, there are 334 patients for whom complete sets of observations (i.e. all relevant outcomes at all time points) are recorded. We use these 334 patients for whom complete data exist as a sample on whom a missingness structure will be applied such that inference made using imputed data can be compared to the original, ‘true’, data. Our interest lies in examining the performance of the multiple imputation methods on data that resemble closely that which would be obtained in a real longitudinal study. As such, we feel that it is more appropriate to apply missingness structures to data from the CARDERA trial rather than simply generating data.

### Structure of Simulated Datasets

Using data on the 334 patients for whom no outcomes are missing, we introduce approximately 20 % missingness, balanced over the four treatment groups, so that imputed outcomes can be compared with known outcomes. This is repeated to create five datasets across which each patient exhibits missing outcomes exactly once and missingness is always balanced by treatment group through stratification. We label the five datasets A, B, C, D, E and Table 1 provides a summary of the datasets with respect to their composition. This is done firstly where outcomes are missing only at 24 months for the selected patients and secondly where outcomes are missing at 12, 18 and 24 months for the same patients. Thus, we have five datasets of each type (missingness at 24 months only and missingness at 12, 18 and 24 months) on which imputation can be performed and compared to the original dataset in which there is no missingness. Missingness is applied completely at random with no conditioning on any patient features or outcomes. This missing completely at random assumption [13] ensures that the ML- and LI-based imputation methods are directly comparable because the sets of assumptions for both are met.

**Table 1:** Table showing the composition of the missing portions of each dataset with respect to treatment group

Treatment group	No. of patients exhibiting missing outcomes in datasets A–E				
	A	B	C	D	E
None	16	17	17	17	16
CSP	16	15	15	15	15
PDN	17	17	17	17	17
Both	18	18	18	18	18
Total	67	67	67	67	66

Each dataset (A–E) features approximately 20 % of outcomes missing at 24 months (and, secondly, at 12, 18 and 24 months)

### Models Used for Estimation

We carried out the multiple imputation techniques described in Sect. 4 and Appendix with these five datasets, with imputations being performed ten times for each of the five datasets. In addition, we imputed missing $\text {ACR}^{(24)}20$ values directly using an auto-regressive logistic regression model under the assumption of a Bernoulli distribution for $\text {ACR}^{(24)}20$ where MLE was used for parameter estimation. We refer to this method of multiple imputation as the ‘direct’ method. For each imputed dataset, we considered two logistic regression models for the estimation of $\mathbb {P}(\text {ACR}^{(24)}20 = 1)$ for each treatment group - an additive model and an interaction model. For notational convenience, we use indices to define treatment groups rather than explicitly defining explanatory variables. The indices *r* and *s* are defined as:

$$\begin{aligned} r = {\left\{ \begin{array}{ll} 1 &{} \text {if patient is prescribed CSP;}\\ 0 &{} \text {otherwise} \end{array}\right. } \end{aligned}$$

and

$$\begin{aligned} s = {\left\{ \begin{array}{ll} 1 &{} \text {if patient is prescribed PDN;}\\ 0 &{} \text {otherwise.} \end{array}\right. } \end{aligned}$$

Then, if we define $\pi _{rs} = \mathbb {P}(\text {ACR}^{(24)}20 = 1|r,s)$, we write the ‘additive’ model as

$$\begin{aligned} \text {log}\left( \frac{\pi _{rs}}{1-\pi _{rs}}\right) = \alpha +\beta _{r}+\gamma _{s} \end{aligned}$$

with the identifiability constraints $\beta _{0} = \gamma _{0} = 0$. The model is described as ‘additive’ because the combined effect of CSP and PDN, compared to MTX only, is obtained through the addition of the parameters $\beta _{r}$ and $\gamma _{s}$. Furthermore, if we define *l* to be an alternative treatment group index such that $l \in \{1, 2, 3, 4\}$ (with treatment group numberings defined as: 1 = “None”, 2 =“CSP”, 3 = “PDN”, 4 = “Both”) then a regression model that includes an interaction to the model for $\pi _{rs}$, which we term the interaction model, can be written as

$$\begin{aligned} \text {log}\left( \frac{\pi _{l}}{1-\pi _{l}}\right) = \phi _{l}, \end{aligned}$$

where $\pi _{l} = \mathbb {P}(\text {ACR}^{(24)}20 = 1|l)$. This model was considered in case the combined effects of CSP and PDN would not be considered as additive.

### Results

We present tables of results that show both the estimated linear predictors for the additive and interaction models, together with estimates of $\mathbb {P}(\text {ACR}^{(24)}20 = 1)$. The multiple imputation and parameter estimation was performed for each of the five datasets (A, B, C, D and E) described in Table 1. In addition, we considered multiple imputation where either missingness occurred at 24 months only or where missingness occurred at each of 12, 18 and 24 months. For brevity, we present sample means of parameter estimates and associated standard errors using imputation results from the five simulated datasets (since results were similar), although full results are available from the authors.

#### Missingness at 24 Months

Table 2 provides a summary of estimated linear predictors (log–odds of the event $\{\text {ACR}^{(24)}20 = 1\}$) and probabilities of the event $\{\text {ACR}^{(24)}20 = 1\}$ calculated using both the additive and interaction prediction models, for each treatment group, where missingness occurred at 24 months only for patients who had non-complete data.

**Table 2:** Table showing the average linear predictor estimates, together with associated standard errors, and estimates of $\mathbb {P}(\text {ACR}^{(24)}20 = 1)$ for the different treatment groups using both the additive and interaction estimation models

Treatment group	Imputation method			
	MLE	LI	DIRECT	TRUE Data
Additive Model: Average Linear Predictor Estimate (Standard error)				
None	$-0.571$ (0.204)	$-0.595$ (0.210)	$-0.540$ (0.219)	$-0.514$ (0.196)
CSP	$-0.380$ (0.205)	$-0.384$ (0.213)	$-0.307$ (0.217)	$-0.322$ (0.199)
PDN	$-0.473$ (0.201)	$-0.515$ (0.208)	$-0.480$ (0.206)	$-0.458$ (0.193)
Both	$-0.283$ (0.197)	$-0.304$ (0.202)	$-0.247$ (0.199)	$-0.265$ (0.187)
Additive Model: Average Estimate of $\mathbb {P}(\text {ACR}^{(24)}20 = 1)$				
None	0.361	0.356	0.368	0.374
CSP	0.406	0.405	0.424	0.420
PDN	0.384	0.374	0.382	0.388
Both	0.430	0.425	0.439	0.434
Interaction Model: Average Linear Predictor Estimate (Standard error)				
None	$-0.374$ (0.232)	$-0.400$ (0.238)	$-0.338$ (0.246)	$-0.316$ (0.222)
CSP	$-0.598$ (0.250)	$-0.601$ (0.260)	$-0.529$ (0.259)	$-0.539$ (0.238)
PDN	$-0.671$ (0.238)	$-0.713$ (0.248)	$-0.687$ (0.247)	$-0.658$ (0.229)
Both	$-0.110$ (0.222)	$-0.132$ (0.226)	$-0.068$ (0.248)	$-0.089$ (0.211)
Interaction Model: Average Estimate of $\mathbb {P}(\text {ACR}^{(24)}20 = 1)$				
None	0.408	0.402	0.416	0.422
CSP	0.355	0.355	0.371	0.368
PDN	0.338	0.329	0.335	0.341
Both	0.473	0.467	0.483	0.478

Results are shown where multiple imputation was performed for all outcomes using maximum likelihood estimation (MLE), for all outcomes using linear increments (LI), direct imputation of $\text {ACR}^{(24)}20$ using ML (DIRECT) and estimates produced using data prior to the application of a missingness structure (TRUE Data). Missing data occurred at 24 months only

**Table 3:** Table summarising the differences between the imputed $\text {ACR}^{(24)}20$ values and the true $\text {ACR}^{(24)}20$ for each imputation method, for those cases where outcomes were missing at 24 months across the ten multiple imputation runs

Difference	Imputation method		
$\text {ACR}^{(24)}20$: Imputed $-$ True	MLE	LI	DIRECT
$-1$	547	614	581
0	2387	2344	2189
+1	406	382	570

Examining Table 2, we see that each imputation method has produced linear predictor and probability estimates that lie reasonably close to those calculated using the true data (column 5 of Table 2). As we would expect, the standard error estimates for estimators produced using imputed data are larger than those produced using the true data. There are no obvious differences in estimation performance, for any method. In addition, the standard error estimates for parameters estimated using data where imputation was performed using linear increments are generally larger than those for parameters where missing data were imputed using maximum likelihood. We might expect this, since fewer assumptions are made in the LI multiple imputation process when compared to the MLE multiple imputation process.

Multiple imputation is often viewed as a method to obtain an unbiased estimate of a population mean or some other population-level parameter. In this work, our aim is to produce a population-level estimate of $\mathbb {P}(\text {ACR}^{(24)}20 = 1)$. At the individual level, the calculation of $\text {ACR}^{(24)}20$ relies directly on accurate estimates of the constituent values for $\text {ACR}^{(24)}20$, outlined in Sect. 2. This places importance on the accuracy of these individual-level values. Table 3 provides a summary of the differences between the true $\text {ACR}^{(24)}20$ values and the imputed $\text {ACR}^{(24)}20$ values aggregated for all ten imputation runs and the three methods of multiple imputation, where outcomes were missing. The results indicate that the MLE- and LI-based methods both predict $\text {ACR}^{(24)}20$ correctly in the majority of cases, with similar proportions of correct predictions (71 % and 70 % of predictions were correct, respectively). The direct imputation method was slightly less successful at correctly predicting $\text {ACR}^{(24)}20$ (66 % of predictions were correct). When direct imputation of $\text {ACR}^{(24)}20$ is performed, it is possible that individual-level predictions of $\text {ACR}^{(24)}20$ may tend to be drawn close to the MLE of the population mean and this could compromise such individual-level predictions. This would be typical of prediction from simple MLE-based models, where shrinkage to the estimate of the population mean is well known [3, 4]. The imputation of each outcome separately, either by MLE or using the LI method may be more appropriate for individual-level predictions.

**Fig. 1:**
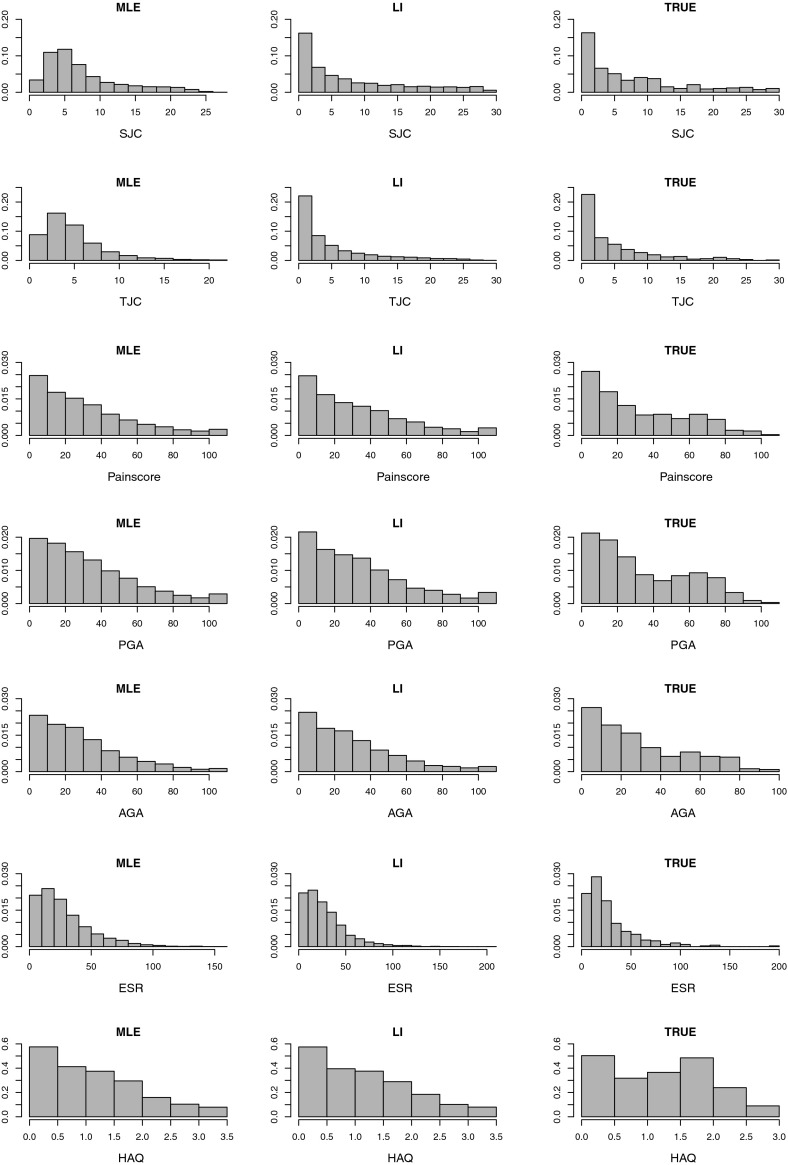
Histograms of the variables at 24 months used to calculate $\text {ACR}^{(24)}20$. ‘TRUE’ denotes the true values, ‘MLE’ denotes values imputed by ML-based models and ‘LI’ denotes values imputed using linear increments models. Missingness was simulated at 24 months only prior to multiple imputation

Figure 1 shows histograms of the outcomes including imputed values, using each imputation method, at 24 months for each outcome used in the calculation of $\text {ACR}^{(24)}20$. In addition, histograms of the true values of the outcomes are shown. The histograms show the estimated distributions of the individual outcomes to be broadly similar for each imputation method, except for the tender and swollen joint counts where the MLE-based method appears to have drawn a relatively large proportion of imputed outcomes close to the observed sample mean outcome, in each case. This might suggest that MLE-based imputation of binomial outcomes is desirable when the aim is to achieve an accurate and precise estimate of the population mean. LI-based imputation is perhaps less likely to provide as precise an estimate of the population mean as the MLE-based methods, although the overall distributional shapes obtained via LI estimation may be more like those seen for the true data, especially for non-normally distributed outcomes.

#### Missingness at 12, 18 and 24 Months

We also considered a situation where the approximately 20 % of patients that have missing outcomes have these outcomes missing at each of 12, 18 and 24 months. To simulate this scenario, missingness was introduced completely at random at 12 months in 20 % of patients. Corresponding patients were then deemed as having ‘dropped-out’ of the trial at 12 months and their outcomes for 18 and 24 months were also deleted.

The outcome of interest remains the value of $\text {ACR}^{(24)}20$ but, in this case, missing outcomes are imputed successively at 12 then 18 then 24 months, using each of the MLE and LI imputation methods. As previously mentioned, imputations were carried out ten times and the estimated linear predictors were combined using Rubin’s rules under the additive and interaction models separately.

**Fig. 2:**
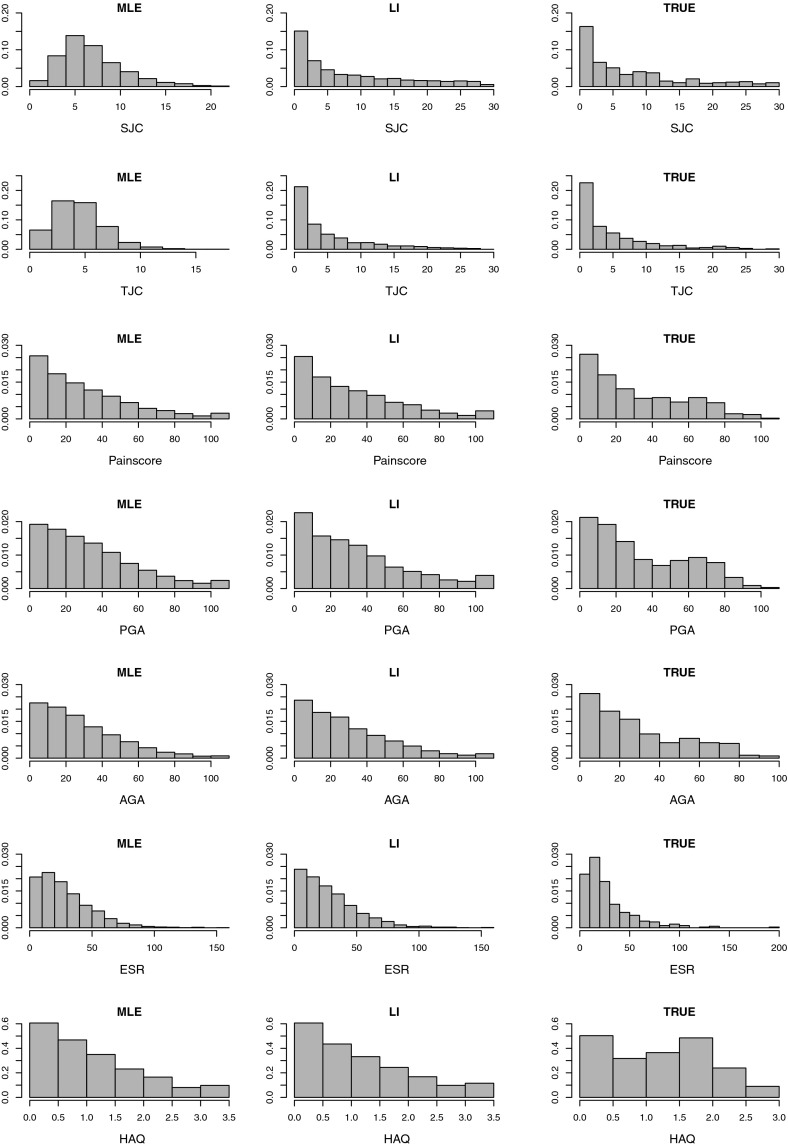
Histograms of the variables at 24 months used to calculate $\text {ACR}^{(24)}20$. ‘TRUE’ denotes the true values, ‘MLE’ denotes values imputed by ML-based models and ‘LI’ denotes values imputed using linear increments models. Missingness was simulated at 12, 18 and 24 months, prior to multiple imputation

Results of these analyses that parallel those for the datasets when missingness is only at 24 months are given in Tables 4, 5 and in Fig. 2. The patterns of results are very similar to those seen when missingness was only at 24 months.

**Table 4:** Table showing the average linear predictor estimates, together with associated standard errors, and estimates of $\mathbb {P}(\text {ACR}^{(24)}20 = 1)$ for the different treatment groups using both the additive and interaction estimation models

Treatment group	Imputation method			
	MLE	LI	DIRECT	TRUE Data
Additive Model: average linear predictor estimate (standard error)				
None	$-0.510$ (0.209)	$-0.586$ (0.221)	$-0.525$ (0.226)	$-0.514$ (0.196)
CSP	$-0.410$ (0.210)	$-0.400$ (0.221)	$-0.341$ (0.229)	$-0.322$ (0.199)
PDN	$-0.395$ (0.202)	$-0.520$ (0.208)	$-0.471$ (0.217)	$-0.458$ (0.193)
Both	$-0.294$ (0.198)	$-0.334$ (0.205)	$-0.288$ (0.209)	$-0.265$ (0.187)
Additive Model: average estimate of $\mathbb {P}(\text {ACR}^{(24)}20 = 1)$				
None	0.375	0.358	0.372	0.374
CSP	0.399	0.401	0.416	0.420
PDN	0.403	0.373	0.384	0.388
Both	0.427	0.417	0.429	0.434
Interaction Model: average linear predictor estimate (standard error)				
None	$-0.329$ (0.239)	$-0.411$ (0.247)	$-0.315$ (0.253)	$-0.316$ (0.222)
CSP	$-0.614$ (0.254)	$-0.593$ (0.263)	$-0.574$ (0.276)	$-0.539$ (0.238)
PDN	$-0.574$ (0.236)	$-0.697$ (0.247)	$-0.685$ (0.253)	$-0.658$ (0.229)
Both	$-0.133$ (0.223)	$-0.179$ (0.23)	$-0.101$ (0.266)	$-0.089$ (0.211)
Interaction Model: average estimate of $\mathbb {P}(\text {ACR}^{(24)}20 = 1)$				
None	0.419	0.399	0.422	0.422
CSP	0.352	0.356	0.361	0.368
PDN	0.360	0.333	0.335	0.341
Both	0.467	0.455	0.475	0.478

Results are shown where multiple imputation was performed for all outcomes using maximum likelihood estimation (MLE), for all outcomes using linear increments (LI), direct imputation of $\text {ACR}^{(24)}20$ using ML (DIRECT) and estimates produced using data prior to the application of a missingness structure (TRUE Data). Missing data occurred at 12, 18 and 24 months

**Table 5:** Table summarising the differences between the imputed $\text {ACR}^{(24)}20$ values and the true $\text {ACR}^{(24)}20$ for each imputation method, for those cases where outcomes were missing at 12, 18 and 24 months across the ten multiple imputation runs

Difference	Imputation method		
$\text {ACR}^{(24)}20$: Imputed $-$ True	MLE	LI	DIRECT
$-1$	651	787	788
0	2120	2053	1826
+1	569	506	726

## Example: Imputation of ACR20 at 24 Months in the CARDERA Trial

In this section, we apply the three methods of imputation described in Sect. 3 to the actual missing values in the CARDERA trial dataset. In addition, we use a chained equations approach, similar to that discussed in [20], to impute each constituent outcome used in the calculation of $\text {ACR}^{(24)}20$ at each time point where a missing value occurs. The chained equations method is flexible, allowing outcomes of different types (both continuous and binary) to be imputed and relaxes the assumption of independence between individual outcomes. The method can be implemented using standard statistical software for MLE-based multiple imputation and can be compared with the other methods for this example.

**Fig. 3:**
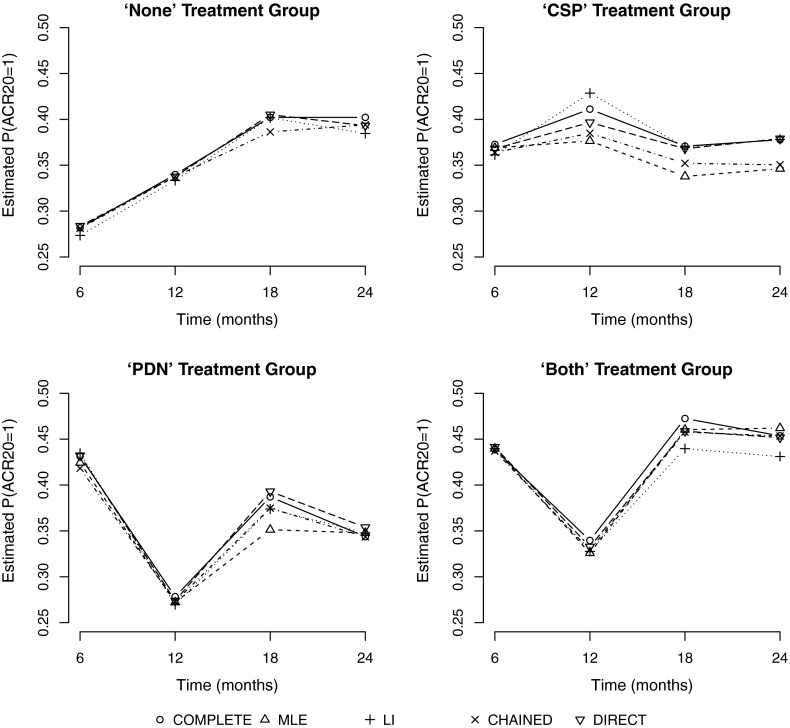
Plot of sample mean estimates of $\mathbb {P}(\text {ACR}^{(24)}20 = 1)$ at each time point (6, 12, 18 and 24 months). Results are shown where multiple imputation has been performed using the ML method (MLE), linear increments method (LI), chained equations method (CHAINED) and direct imputation method (DIRECT). Results for the complete cases data (Complete) are also shown. Separate plots are shown for each of the four trial arms

The true missingness mechanism of the CARDERA data is unknown, but it is unlikely that missing data are missing completely at random. Results are presented for both the additive and interaction models (with respect to trial arm) in Table 6, in a similar manner to the results presented in Tables 2 and 4. We note that, since $\text {ACR}^{(24)}20$ is genuinely missing for these patients, there is no column in Table 6 to show the ‘TRUE’ $\text {ACR}^{(24)}20$ values. Estimates from a complete cases analysis are also shown. For a comparison over time, Fig. 3 shows plots of mean estimates of $\mathbb {P}(\text {ACR}^{(24)}20=1)$ (obtained using Rubin’s rules) at each time point (6, 12, 18 and 24 months) for each imputation method, compared to equivalent estimates formed using the complete cases data. Plots are shown separately for each trial arm.

**Table 6:** Table showing the average linear predictor estimates, together with associated standard errors, and estimates of $\mathbb {P}(\text {ACR}^{(24)}20 = 1)$ for the different treatment groups using both the additive and interaction estimation models

Group	Imputation Method				
	MLE	LI	CHAINED	DIRECT	COMPLETE
Additive Model: linear predictor estimate (standard error)					
None	$-0.608$ (0.180)	$-0.623$ (0.192)	$-0.591$ (0.183)	$-0.553$ (0.200)	$-0.540$ (0.186)
CSP	$-0.482$ (0.174)	$-0.486$ (0.180)	$-0.452$ (0.176)	$-0.380$ (0.193)	$-0.353$ (0.185)
PDN	$-0.511$ (0.175)	$-0.545$ (0.180)	$-0.480$ (0.176)	$-0.481$ (0.166)	$-0.505$ (0.183)
Both	$-0.385$ (0.179)	$-0.407$ (0.184)	$-0.341$ (0.179)	$-0.308$ (0.174)	$-0.318$ (0.180)
Additive Model: estimate of $\mathbb {P}(\text {ACR}^{(24)}20 = 1)$					
None	0.353	0.349	0.356	0.365	0.368
CSP	0.382	0.381	0.389	0.406	0.413
PDN	0.375	0.382	0.367	0.382	0.376
Both	0.405	0.400	0.416	0.424	0.421
Interaction Model: linear predictor estimate (standard error)					
None	$-0.442$ (0.205)	$-0.493$ (0.215)	$-0.427$ (0.205)	$-0.435$ (0.222)	$-0.396$ (0.213)
CSP	$-0.647$ (0.204)	$-0.614$ (0.209)	$-0.614$ (0.206)	$-0.495$ (0.216)	$-0.499$ (0.217)
PDN	$-0.683$ (0.211)	$-0.680$ (0.210)	$-0.649$ (0.213)	$-0.602$ (0.195)	$-0.647$ (0.215)
Both	$-0.225$ (0.198)	$-0.281$ (0.200)	$-0.183$ (0.198)	$-0.194$ (0.202)	$-0.186$ (0.204)
Interaction Model: estimate of $\mathbb {P}(\text {ACR}^{(24)}20 = 1)$					
None	0.391	0.379	0.395	0.393	0.402
CSP	0.344	0.351	0.351	0.379	0.378
PDN	0.335	0.336	0.343	0.354	0.344
Both	0.444	0.430	0.454	0.452	0.454

Multiple imputation has been used to predict actual missing $\text {ACR}^{(24)}20$ values from the CARDERA trial. Results are shown where multiple imputation was performed for all outcomes using maximum likelihood estimate (MLE), for all outcomes using linear increments (LI), for all outcomes using a chained equations approach (CHAINED) and via direct imputation of $\text {ACR}^{(24)}20$ using maximum likelihood (DIRECT). As a comparison, results using complete cases only (COMPLETE) are also shown

Table 6 shows that the four methods perform similarly with regard to the prediction of $\mathbb {P}(\text {ACR}^{(24)}20 = 1)$ using both the additive and interaction models. The similarity of the MLE and LI methods is encouraging and suggests that each of these methods would be applicable for the multiple imputation of $\text {ACR}^{(24)}20$, subject, as always, to their respective assumptions regarding the pattern(s) of missingness within the dataset. We note that the chained equations approach, which does not necessarily assume independence amongst the different composite outcomes, has resulted in point estimates and standard error estimates that lie close to those from both the MLE and LI approaches. This suggests that the assumption of independence amongst outcomes in the LI and MLE approaches seems to be plausible in this example. In principle, a multivariate approach based on LI models [1] is possible but this would require bespoke software.

Comparing the imputed data to the complete cases estimates (Fig. 3), we see that the general patterns over time are similar between the complete cases estimates and results from each imputation method and across all four trial arms with the exception of the CSP group, for which discrepancies are more noticeable. Farewell [8] showed that population means estimated from a GEE with an AR(1) working covariance model are intermediate to the complete case and linear increments estimates. For the kind of moderate-to-strong correlation typical of longitudinal data such as these, we might therefore expect the ML estimates also to be intermediate, but likely, nearer to the LI extreme. This pattern is consistent with that seen in Fig. 3 and Table 6.

In Table 6, we see that the standard error estimates for the linear predictors are comparable between the different imputation approaches and the complete case standard error estimate, for both the additive and interaction models. This suggests that overall, for this example, the different approaches yield robust and comparably precise estimates.

## Discussion

We have explored the use of MLE- and LI-based methods for multiple imputation of a binary composite outcome in a longitudinal clinical trial. Both methods were used to impute values for the components of the composite outcome and we compared these approaches and direct MLE-based imputation of the composite outcome.

The LI estimates of means used for multiple imputation are non-parametric and do not depend on distributional assumptions. Conversely, the ML population mean estimates depend on the distributional assumptions made. The LI estimates might, therefore, be expected to be more robust if the distributional assumption is incorrect and the MLEs more efficient if the assumption is correct. Nevertheless, the distributional assumptions are required in both methods for the repeat imputation of missing outcomes.

In general, the missingness structure of any dataset will never be known exactly. All imputation methods rely on assumptions regarding the missingness process and such assumptions are untestable. As a result, we did not attempt to explore the methods under strictly defined missingness structures. In practice, sensible and practical intuition would be important in defining an appropriate missingness structure, and we would recommend that both MLE- and LI-based imputation models be explored and their results compared under any defined missingness structure. Some researchers have used an LI approach to the multiple imputation of missing values as a comparison to an MLE-based chained equations approach in the analysis of a clinical trial [11].

For a single outcome, it is well known that multiple imputation using MLE-based models is an appropriate method to obtain an unbiased estimate of the population mean, under the missing at random assumption that missingness does not depend on unobserved data. We generated missingness under this assumption and observed comparable performance of the two methods. Comparable performance for estimation of mean outcomes was also seen using direct imputation of the composite outcome.

The imputation of individual outcomes relies on modelling assumptions for each individual outcome. If such modelling assumptions were correct, then this could result in the imputation of individual outcomes being preferable to the direct imputation of the composite outcome, because accurate accounting for missingness in both the individual outcomes and composite outcome would be made, resulting in a more complete longitudinal dataset for analysis. However, we note that the adoption of modelling assumptions for each individual outcome naturally makes the multiple imputation of the individual outcomes reliant on more modelling assumptions than direct imputation of the composite outcome.

The similar performance of the component-based multiple imputation using LI methods and MLE methods, in simulations when missingness is completely at random and for the actual CARDERA trial data, which would be expected to have a more general pattern of missingness, suggests that LI-based multiple imputation may be a useful tool for the validation of MLE-based multiple imputation for missing composite outcomes. Differing results might suggest that distributional assumptions used in MLE-based multiple imputation models should be re-examined. Agreement between the methods would be reassuring in this regard. Irresolvable disagreement would motivate further discussion of the relative plausibility of the assumed missing data mechanisms or statistical modelling assumptions.

## Appendix: Imputation Algorithms

### Maximum Likelihood Model

We assume a normal distribution for all outcomes except for the TJC and SJC outcomes, for which we assume binomial distributions. The initial ML imputation process for the normally distributed outcomes that contribute to ACR20 can be described as follows.

1. Suppose that $g(Y_{ij})$ denotes the transformed outcome of interest for the *i*th individual at time point $t_{j}$, where $g(Y_{ij})$ is assumed to be normally distributed. A model of the form given in (3) is defined at time points $t_{j}$, $j\in \{2, 3, 4, 5\}$. That is:
  $$\begin{aligned} g(Y_{ij}) = \alpha _{j}+\beta _{j}g(y_{ij-1})+\varvec{\gamma }^{T}\mathbf {x}_{i}+\epsilon _{ij}. \end{aligned}$$
  Here $g(y_{ij-1})$ denotes the observed, transformed outcome at the previous time point and $\mathbf {x}_{i}$ denotes as vector of indicator variables pertaining to treatment group of the form:
  $$\begin{aligned} \mathbf {x}_{i} = \left( I({\{\mathrm{Group}_{i} = `CSP\text {'}\}}), I({\{\mathrm{Group}_{i} = `PDN\text {'}\}}), I({\{\mathrm{Group}_{i} = `Both\text {'}\}})\right) ^{T} \end{aligned}$$
  for the *i*th individual, $\text {Group}_{i}$ denotes the treatment group of the *i*th individual. $\epsilon _{ij}\sim \mathcal {N}(0,\sigma _{j}^{2})$ is a mean-zero error term. The term $I(\{E\})$ denotes an indicator function for the event *E*.
2. The model in Step 1 is fitted using complete (non-missing) cases at each time point ($t_{1}, t_{2}, t_{3}, t_{4}, t_{5}$) and MLE methods. Corresponding parameter estimates $(\hat{\alpha }_{j}, \hat{\beta }_{j}, \hat{\varvec{\gamma }}_{j}^{T},\hat{\sigma }_{j}^{2})^{T}$ are obtained, together with their associated asymptotic distributions.
3. A random draw is performed from each of the asymptotic distributions of $(\hat{\alpha }_{j}, \hat{\beta }_{j}, \hat{\varvec{\gamma }}_{j}^{T},\hat{\sigma }_{j}^{2})^{T}$, to reflect the uncertainty in these parameter estimates. This creates a new set of parameter estimates, which we denote $(\tilde{\alpha }_{j}, \tilde{\beta }_{j}, \tilde{\varvec{\gamma }}_{j}^{T},\tilde{\sigma }_{j}^{2})^{T}$.
4. Using the new parameter estimates, drawn in Step 3, we compute an estimated mean transformed outcome variable at $t_{j}$ for the *i*th individual (where the corresponding outcome variable is missing), given by
  $$\begin{aligned} \tilde{g}(y_{ij}) = \tilde{\alpha }_{j}+\tilde{\beta }_{j}g(y_{ij-1})+\tilde{\gamma }_{j}^{T}\mathbf {x}_{i}. \end{aligned}$$
5. We use $\tilde{g}(y_{ij})$ as a proxy for $\mathbb {E}(g(Y_{ij}))$ and $\tilde{\sigma }^{2}_{j}$ as a proxy for $\text {Var}(g(Y_{ij}))$. We draw a value from a $\mathcal {N}(\tilde{g}(y_{ij}),\tilde{\sigma }^{2}_{j})$ distribution and denote this draw $g^{\text {(imp)}}(y_{ij})$.
6. The value $g^{\text {(imp)}}(y_{ij})$ is transformed using the inverse function $g^{-1}()$ to obtain an estimate of the outcome for the *i*th individual at time point $t_{j}$. This value is imputed into the dataset in place of the *i*th individual’s missing observation at time point $t_{j}$.
7. Steps 3–6 are repeated $M \in \mathbb {N}$ times to create values for *M* imputed datasets to be analysed.

For the TJC and SJC, we use logistic regression models (because we have binomial outcomes), and the following algorithm is used:

1. Suppose that $\pi (Y_{ij})$ denotes the probability of the event of interest (i.e. a tender or swollen joint) for the *i*th individual at time point $t_{j}$, where $Y_{ij}$ is the tender or STC for the *i*th individual at time point $t_{j}$. A model of the form given in (3) is defined at time points $t_{j}$, $j\in \{2, 3, 4, 5\}$. That is:
  $$\begin{aligned} \log \left( \frac{\pi (Y_{ij})}{1-\pi (Y_{ij})}\right) = \alpha _{j}+\beta _{j}y_{ij-1}+\varvec{\gamma }^{T}\mathbf {x}_{i}. \end{aligned}$$
  Here $y_{ij-1}$ denotes the observed tender/swollen joint count at the previous time point and $\mathbf {x}_{i}$ denotes as vector of indicator variables pertaining to treatment group of the form:
  $$\begin{aligned} \mathbf {x}_{i} = \left( I({\{\text {Group}_{i} = `CSP\text {'}\}}), I({\{\text {Group}_{i} = `PDN\text {'}\}}), I({\{\text {Group}_{i} = `Both\text {'}\}})\right) ^{T} \end{aligned}$$
  for the *i*th individual, $\text {Group}_{i}$ denotes the treatment groups of the *i*th individual.
2. The model in Step 1 is fitted to complete (non-missing) cases at each time point ($t_{1}, t_{2}, t_{3}, t_{4}, t_{5}$) using MLE methods, under the assumption that the joint count is binomially distributed. Corresponding parameter estimates $(\hat{\alpha }_{j}, \hat{\beta }_{j}, \hat{\varvec{\gamma }}_{j}^{T})^{T}$ are obtained, together with their associated asymptotic distributions.
3. A random draw is performed from each of the asymptotic distributions of $(\hat{\alpha }_{j}, \hat{\beta }_{j}, \hat{\varvec{\gamma }}_{j}^{T})^{T}$, to reflect the uncertainty in these parameter estimates. This creates a new set of parameter estimates, which we denote $(\tilde{\alpha }_{j}, \tilde{\beta }_{j}, \tilde{\varvec{\gamma }}_{j}^{T})^{T}$.
4. An estimate for the probability of a damaged joint, for the *i*th individual at time $t_{j}$ is computed as
  $$\begin{aligned} \tilde{\pi }(y_{ij}) = \frac{\exp \left( \tilde{\alpha }_{j}+\tilde{\beta }_{j}y_{ij-1}+\tilde{\varvec{\gamma }}^{T}\mathbf {x}_{i}\right) }{1+\exp \left( \tilde{\alpha }_{j}+\tilde{\beta }_{j}y_{ij-1}+\tilde{\varvec{\gamma }}^{T}\mathbf {x}_{i}\right) } \end{aligned}$$
5. Using $\tilde{\pi }(y_{ij})$ as a proxy for the ‘true’ probability of a tender/swollen joint at time point $t_{j}$, we make a random draw from a $\text {Bin}(28,\tilde{\pi }(y_{ij}))$ distribution. We denote this drawn value $y^{\text {(imp)}}_{ij}$. The value $y^{\text {(imp)}}_{ij}$ is imputed into the dataset in place of the *i*th individual’s missing observation at time point $t_{j}$.
6. Steps 3–5 are repeated $M \in \mathbb {N}$ times to create values for *M* imputed datasets to be analysed.

### Linear Increments Model

The idea behind the LI method is to model the increments of a longitudinal process: $Y_{ij}-Y_{ij-1}$. Although the LI estimates themselves are non-parametric (Eq. 2), we require an assumption for the distribution of the outcomes so that random draws can be made (although parameters need not be estimated using a method that relies on distributional assumptions). Clearly, if we assume that $g(Y_{ij})$ is normally distributed, then it follows that the increment $g(Y_{ij})-g(Y_{ij-1})$ is also normally distributed. This is used for most of the outcomes involved in the calculation of ACR20. However, the tender and swollen joint counts have binomial distributions, and so the increment $Y_{ij}-Y_{ij-1}$ has no standard distributional form. To alleviate this problem, we consider $g(Y_{ij})$ to be the empirical logit transformation of the joint count $Y_{ij}$ where $Y_{ij} \sim \text {Bin}(28, \pi _{ij})$. That is,

$$\begin{aligned} g(Y_{ij})= & {} \text {log}\left( \frac{Y_{ij}+0.5}{28-Y_{ij}+0.5}\right) . \end{aligned}$$

For all other outcomes that make up ACR20, the transformation is given by $g(Y_{ij}) = (Y_{ij})^{\frac{1}{2}}$. The imputation process for each outcome that is used to form ACR20 is described below.

1. We define an LI model of the form
  $$\begin{aligned} \mathbb {E}(g(Y_{ij})-g(Y_{ij-1})) = \alpha _{j}+\beta g(y_{ij-1})+\varvec{\gamma }^{T}\mathbf {x}_{i}. \end{aligned}$$
  Here $g(y_{ij-1})$ denotes the observed, transformed outcome at the previous time point and $\mathbf {x}_{i}$ denotes as vector of indicator variables pertaining to treatment group of the form:
  $$\begin{aligned} \mathbf {x}_{i} = \left( I({\{\text {Group}_{i} = `CSP\text {'}\}}), I({\{\text {Group}_{i} = `PDN\text {'}\}}), I({\{\text {Group}_{i} = `Both\text {'}\}})\right) ^{T} \end{aligned}$$
  for the *i*th individual, $\text {Group}_{i}$ denotes the treatment group of the *i*th individual.
2. The model defined in Step 1 is fitted using generalised estimating equations (using the method outlined in Sect. 3). From the model fit, we obtain estimated model parameters $(\hat{\alpha }_{j}, \hat{\beta }_{j}, \hat{\varvec{\gamma }}_{j}^{T})^{T}$, together with an estimated robust variance–covariance matrix for $(\hat{\alpha }_{j}, \hat{\beta }_{j}, \hat{\varvec{\gamma }}_{j}^{T})^{T}$, which we term $\hat{\Sigma }_{\mathcal {R}}$.
3. To account for the uncertainty in the estimation of $(\hat{\alpha }_{j}, \hat{\beta }_{j}, \hat{\varvec{\gamma }}_{j}^{T})^{T}$, we re-draw the model parameters at random from a multivariate $\mathcal {N}((\hat{\alpha }_{j}, \hat{\beta }_{j}, \hat{\varvec{\gamma }}_{j}^{T})^{T},\hat{\Sigma }_{\mathcal {R}})$ distribution, yielding corresponding updated parameter estimates, which we denote $(\tilde{\alpha }_{j}, \tilde{\beta }_{j}, \tilde{\varvec{\gamma }}_{j}^{T})^{T}$.
4. Using the new parameter estimates, drawn in Step 3, we compute an estimated increment value from $t_{j-1}$ to $t_{j}$ for the outcome variable in the *i*th individual (where the corresponding outcome variable is missing), given by
  $$\begin{aligned} \tilde{\Delta }(g(y_{ij}))= \tilde{\alpha }_{j}+\tilde{\beta }_{j}g(y_{ij-1})+\tilde{\gamma }_{j}^{T}\mathbf {x}_{i}. \end{aligned}$$
5. We calculate $s^{2}_{j-1,j}$ as the sample variance of the difference in the observed, transformed values ($g(y_{ij})-g(y_{ij-1})$) between time points $t_{j-1}$ and $t_{j}$. Then, a random value is drawn from a $\mathcal {N}(\tilde{\Delta }(g(y_{ij})),s^{2}_{j-1,j})$ distribution. We call this drawn value $\Delta ^{\text {(imp)}}(g(y_{ij}))$ and define it to be an imputed value for the difference in transformed outcome from time point $t_{j-1}$ to $t_{j}$ for the *i*th individual.
6. Using the imputed increment values from Step 5, an estimated of the missing transformed outcome at time point $t_{j}$ is given by
  $$\begin{aligned} g^{\text {(imp)}}(y_{ij}) = g(y_{ik})+\sum _{l=k+1}^{j}\Delta ^{(\text {imp})}(g(y_{il})). \end{aligned}$$
  Here, we assume that the outcome, for individual *i*, is observed up to time $t_{k}$ for some $k \in \mathcal {N}, k<j$. The value $g^{\text {(imp)}}(y_{ij})$ is transformed to the original scale, using the inverse function $g^{-1}()$, to produce an estimated value $y^{\text {(imp)}}_{ij}$ that is imputed into the original dataset to replace the *i*th individual’s missing observation at time point $t_{j}$.
7. Steps 1–6 are repeated $M \in \mathbb {N}$ times, for each outcome, to create values for *M* imputed datasets to be analysed.
